# Low CP Overhead Waveform Design for Multi-Path Channels with Timing Synchronization Error

**DOI:** 10.3390/s22155772

**Published:** 2022-08-02

**Authors:** Jing Chen, Baobing Wang, Jianzhong Guo, Xin Shan, Dejin Kong

**Affiliations:** 1 Electronic Information School, Wuhan University, Wuhan 430072, China; chen.j@whu.edu.cn (J.C.); sx@whu.edu.cn (X.S.); 2 School of Electronic and Electrical Engineering, Wuhan Textile University, Wuhan 430200, China; 2015363051@mail.wtu.edu.cn (B.W.); jianzg@whu.edu.cn (J.G.)

**Keywords:** OFDM, symbol repetition, multi-path channel, timing synchronization error, cyclic prefix

## Abstract

In classical orthogonal frequency division multiplexing (OFDM) systems, inserting the cyclic prefix (CP) is necessary before each symbol to overcome the multi-path effect, which, however, occupies numerous time-frequency radio resources, resulting in hampered spectrum efficiency. To address this issue, in this paper, symbol repetition aware OFDM (SR-OFDM) is developed to lower the overhead of CP. In the proposed SR-OFDM, multiple symbols share the same CP with which we examine that the multi-path channels can also be overcome by a simple single-tap equalization without causing any interference. Moreover, after the discrete Fourier transform at the receiver, different symbols are proved to be separated in the time domain, which is beneficial for lowering the demodulation complexity. Furthermore, it is revealed that the above conclusions still hold even under timing synchronization errors, which makes the proposed SR-OFDM favorable in real systems. Extensive simulations validate the efficacy of our proposed SR-OFDM system under the multi-path channels with or without timing synchronization errors.

## 1. Introduction

In recent decades, the effective method of improving the data rate has been to increase the system’s bandwidth in wireless communication networks. More precisely, the bandwidths in 3G, 4G, and 5G are up to 5 MHz, 20 MHz, and 400 MHz, respectively [1,2,3]. In general, the increase of system bandwidth results in the decrease of the sampling period [4], which in turn makes the multi-path effect become more and more severe. To overcome the multi-path effect, cyclic prefix (CP) combined with orthogonal frequency division multiplexing (OFDM) is extremely pervasive because of its capability of converting the multi-tap time–domain channel into a single-tap frequency coefficient, reducing the complexity of signal processing significantly. Therefore, CP-OFDM is widely adopted in the physical layers of many communication standards [5,6]. However, CP is a redundancy essentially obtained by duplicating the tail of each OFDM signal [7], which lowers the spectral efficiency greatly. For instance, the overhead of CP accounts for 20% of time-frequency resources in IEEE 802.11a [8]. Therefore, it is of vital importance to lower the overhead of CP while maintaining low complexity of signal detection in wireless communications.

So far, several schemes have been presented to lower the CP overhead. In [9], to shorten CP, a precoding matrix was acquired by building a sum-rate optimization problem to deal with the inter-user interference. In [10], an iterative receiver was proposed by utilizing soft decision feedback to eliminate the interference caused by shortening the CP. In [11], the authors presented an equalization algorithm with symbol cyclic shift to shorten the CP by using stored feedback equalization at the receiver. In [12], based on the overlapping minimum mean square error (MMSE) equalization, the authors proposed a CP-free method for OFDM systems, which performed well at the region of the low signal-to-noise ratio (SNR). Although these wors were devoted to shortening CP, the problem of the remaining interference among symbols is still a non-negligible obstacle, especially at high SNR. What is more, the resulting complexity of signal processing is increased considerably. In fact, the CP overhead can be reduced efficiently via the intuitive idea of reducing the subcarrier spacing at the cost of increasing the sensitivity to the phase noise or frequency offset. In [13,14], the vector OFDM system was presented by adopting a unit matrix for precoding multiple symbols. Since one data block consisting of multiple symbols only includes one CP, the CP overhead can be reduced significantly in the vector OFDM system. However, although there exists no inter-symbol interference, vector OFDM does not consider the complexity of symbol demodulation at the receiver, and all symbols in one block have to be performed jointly.

In this paper, the symbol repetition aware OFDM (SR-OFDM), in which multiple symbols share the same CP and there exists no inter-symbol interference, is developed to lower the overhead of CP. In addition, the received signals of different symbols can be separated in the time domain after the discrete Fourier transform (DFT), and single-tap equalization can be adopted at the receiver, which is beneficial for lowering the demodulation complexity. Furthermore, compared with [15] (our previous work), we extend the influencing factors in SR-OFDM, especially adding a timing synchronization error. It is revealed that when the symbol timing error is within the CP, symbol demodulation with a single-tap equalizer can be performed without causing any inter-symbol interference. Note that the CP length subtracting the timing error is required to be larger than the maximum channel delay. The robustness to the timing error releases the dependence on time synchronization accuracy, which makes our devised SR-OFDM more favorable in practice. For simplicity, main novelties and contributions of this paper are summarized as follows.

- A novel waveform, i.e., SR-OFDM, is developed, aiming to lower the overhead of CP, whilst satisfying the orthogonality of a multi-carrier modulation system.
- Under the designed paradigm, we mathematically show that the multi-path channels can be overcome via the single-tap equalizer without any interference, even under a timing synchronization error.
- With a rigorous complexity analysis, it is demonstrated that the CP overhead is reduced significantly by the proposed SR-OFDM at the cost of an acceptable complexity.
- Two estimation methods are devised for SR-OFDM together with the theoretical analysis on its key performance features, such as, spectral efficiency, peak to average power ratio (PAPR), and complexity.

The remainder of this paper is organized as follows. In Section 2, the novel waveform, SR-OFDM, is presented, including the design of the transmitter and the symbol demodulation at the receiver. Subsequently in Section 3, we present the robustness of SR-OFDM to a timing synchronization error, as well as two methods in regard to the estimation for the channel and timing synchronization error. In Section 4, the spectral efficiency, complexity, and PAPR of SR-OFDM are analyzed and compared with the classical OFDM, respectively. Then, the numerical results are provided in Section 5, followed by the conclusion and future work in Section 6.

For convenience, Table 1 gives several notations used throughout this paper.

## 2. Proposed SR-OFDM

The proposed SR-OFDM in this paper is based on the symbol repetition in OFDM, wherein one piece of data is transmitted repeatedly at one subcarrier for *R* symbol durations. To guarantee the data rate, R−1 other symbols are transmitted simultaneously at the same subcarrier during the same time duration, and phase factors are required to maintain the orthogonality of different symbols. Compared with the classical OFDM system, *R* symbols only occupy one CP in SR-OFDM, hence the CP overhead of SR-OFDM is 1/R of OFDM.

### 2.1. SR-OFDM Transmitter

**Figure 1:**
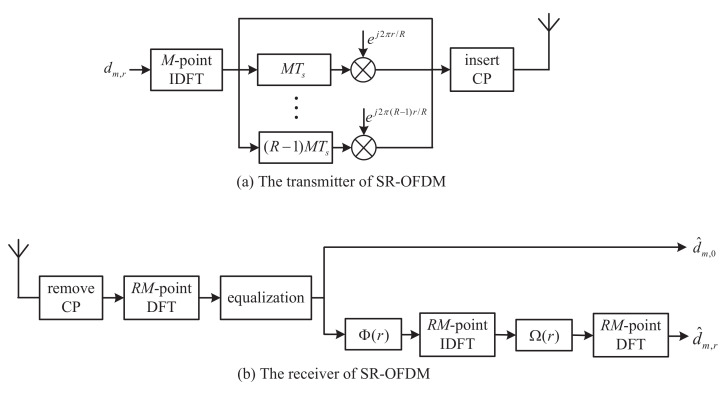
The system diagram of SR-OFDM.

Figure 1a shows the SR-OFDM transmitter equipped with *M* subcarriers, including *R* branches for *R* independent data streams. The *r*-th branch is used to transmit the symbol $d_{m,r}$, where *m* is the index of the subcarrier. For each subcarrier, *R* symbols are transmitted simultaneously during *R* symbols duration, i.e., RT, where *T* is the symbol duration and 1/T is the subcarrier spacing. For the sampling period of $T_{s}=T/M$, the time duration RT corresponds to RM samples. Therefore, when the CP overhead is not considered, SR-OFDM will have the same data rate as the classical OFDM system.

**Figure 2:**
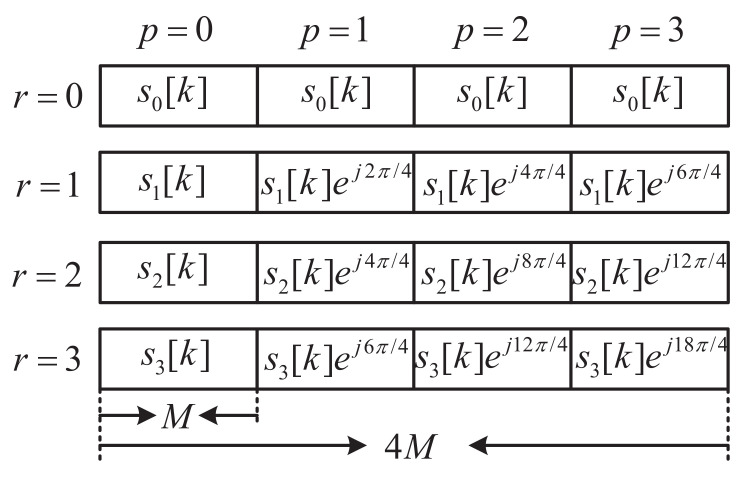
Signal design of ${\bar{s}}_{r}\left[k\right]$, taking R=4 as an example.

For r∈[0,R−1], after the inverse discrete Fourier transform (IDFT) on $d_{m,r},m\;=\;0,1,\ldots ,$ M−1, it can be obtained

(1) $$\begin{array}{c} s_{r}\left[k\right]=\;\;\frac{1}{\sqrt{M}}\;\;\sum_{m=0}^{M-1}d_{m,r}e^{j2\pi mk/M}, \end{array}$$

where k=0,1,…,M−1. Then, the signals $s_{r}\left[k\right]$ are repeated for *R* times. For the *p*-th repetition with p∈[0,R−1], $s_{r}\left[k\right]$ is multiplied by the phase coefficient $e^{j2\pi rp/R}$, as shown in Figure 2. Then, for r∈[0,R−1], the signal is obtained as

(2) $$\begin{array}{c} {\bar{s}}_{r}\left[k\right]=s_{r}\left[k\right]e^{j2\pi rp/R}, \end{array}$$

where $k=pM+\tilde{k},\;p=0,1,\ldots ,R-1\;\mathrm{and}\;\tilde{k}=0,1,\ldots ,M-1$. Next, CP is inserted by the following equation,

(3) $$\begin{array}{c} {\tilde{s}}_{r}\left[k\right]\;\;=\;\;\left\{\begin{array}{cc} \;{\bar{s}}_{r}\left[RM\;+\;k\right], & k\;=\;-\;L_{cp},-\;L_{cp}\;+\;1,\ldots ,-\;1,\\ \;{\bar{s}}_{r}\left[k\right], & k\;=\;0,1,\ldots ,RM\;-\;1, \end{array}\right. \end{array}$$

where $L_{cp}$ is the length of CP. Note that the length of CP should be larger than the maximum channel delay to eliminate the interference among symbols [16].

Finally, the baseband equivalent transmitted signal of SR-OFDM is written as

(4) $$\begin{array}{c} \tilde{s}\left[k\right]=\sum_{r=0}^{R-1}{\tilde{s}}_{r}\left[k\right]. \end{array}$$

### 2.2. SR-OFDM Receiver

Denote a multi-path channel as h[k] with $k=0,1,\ldots ,L_{h}$, where $L_{h}$ is the maximum channel delay. Then, the received signal is written as

(5) $$\begin{array}{cc} r[k] & =\sum_{l=0}^{L_{h}-1}h\left[l\right]\tilde{s}\left[k-l\right]+\eta \left[k\right], \end{array}$$

where $k=-L_{cp},-L_{cp}+1,\ldots ,RM+L_{h}-2$, and η[k] stands for the noise that satisfies the Gaussian distribution [17,18,19].

As shown Figure 1b, CP is removed from the signal r[k], and it is written as

(6) $$\begin{array}{cc} \tilde{r}\left[k\right] & =\sum_{l=0}^{L_{h}-1}h\left[l\right]\tilde{s}\left[k-l\right]+\eta \left[k\right], \end{array}$$

where k=0,1…,RM−1.

In the following, the signals of different $d_{m,r}\left(r=0,1,\ldots ,R-1\right)$ can be separated via n RM-point discrete Fourier transform (DFT), as presented below. The signal separation for different r∈[0,R−1] will lower the demodulation complexity greatly. After DFT on $\tilde{r}\left[k\right]$, it is obtained as

(7) $$\begin{array}{cc} y_{\tilde{m}} & =\frac{1}{\sqrt{RM}}\sum_{k=0}^{RM-1}\tilde{r}\left[k\right]e^{-j2\pi \tilde{m}k/RM}. \end{array}$$

Substituting (4) and (6) into (7), it is obtained as

(8) $$\begin{array}{cc} y_{\tilde{m}} & \;\;=\;\;\frac{1}{\sqrt{R\;M}}\;\sum_{k=0}^{RM-1}\sum_{l=0}^{L_{h}-1}h\left[l\right]\tilde{s}\left[k-l\right]e^{-j2\pi \tilde{m}k/RM}\;+\;\eta_{\tilde{m}}\\ \; & \;\;=\;\;\frac{1}{\sqrt{R\;M}}\;\sum_{k=0}^{RM-1}\sum_{r=0}^{R-1}\sum_{l=0}^{L_{h}-1}h\left[l\right]{\tilde{s}}_{r}\left[k-l\right]e^{-j2\pi \tilde{m}k/RM}\;+\;\eta_{\tilde{m}}\\ \; & \;\;=\;\;\frac{1}{\sqrt{R\;M}}\;\;\;\;\sum_{k=-l}^{RM-1-l}\sum_{r=0}^{R-1}\sum_{l=0}^{L_{h}-1}h\left[l\right]{\tilde{s}}_{r}\left[k\right]e^{-j2\pi \tilde{m}\left(k+l\right)/RM}\;+\;\eta_{\tilde{m}}\\ \; & \;\;=\;\;\frac{1}{\sqrt{R\;M}}\;\;\sum_{r=0}^{R\;-\;1}\;\;\left[\sum_{l=0}^{L_{h}-1}\;\;\;h\left[l\right]e^{-j2\pi \tilde{m}l/RM}\;\right]\;\;\sum_{k=-l}^{RM-1-l}\;\;\;\;\;\;\;{\tilde{s}}_{r}\left[k\right]e^{-j2\pi \tilde{m}k/RM}\;\;+\;\eta_{\tilde{m}}, \end{array}$$

where $\tilde{m}=0,1\ldots ,RM-1$ and the noise term $\eta_{\tilde{m}}$ can be obtained by

(9) $$\begin{array}{c} \eta_{\tilde{m}}=\frac{1}{\sqrt{RM}}\sum_{k=0}^{RM-1}\eta \left[k\right]e^{-j2\pi \tilde{m}k/RM}. \end{array}$$

Due to the fact that ${\tilde{s}}_{r}\left[k\right]$ is a signal with the circular structure according to (3), Equation (8) can be rewritten as

(10) $$\begin{array}{cc} y_{\tilde{m}} & =\frac{1}{\sqrt{RM}}\sum_{r=0}^{R-1}\left[\sum_{l=0}^{L_{h}-1}h\left[l\right]e^{-j2\pi \tilde{m}l/RM}\right]\\ & \;\;\;\;\;\;\;\;\;\;\;\times \sum_{k=0}^{RM-1}{\tilde{s}}_{r}\left[k\right]e^{-j2\pi \tilde{m}k/RM}+\eta_{\tilde{m}}, \end{array}$$

It is obvious that the distribution of $\eta_{\tilde{m}}$ is the same as that of η[k]. In addition, it can be easily observed that the term $\sum_{l=0}^{L_{h}-1}h\left[l\right]e^{-j2\pi \tilde{m}l/RM}$ in (10) is the channel frequency response of h[k]. For the sake of simplicity, we denote $\sum_{l=0}^{L_{h}-1}h\left[l\right]e^{-j2\pi \tilde{m}l/RM}$ by $H_{\tilde{m}}$. Then, substituting (2) into (8), $y_{\tilde{m}}$ can be rewritten as

(11) $$\begin{array}{cc} y_{\tilde{m}} & =\frac{H_{\tilde{m}}}{\sqrt{RM}}\sum_{r=0}^{R-1}\sum_{k=0}^{RM-1}{\tilde{s}}_{r}\left[k\right]e^{-j2\pi \tilde{m}k/RM}+\eta_{\tilde{m}}\\ \; & =\frac{H_{\tilde{m}}}{\sqrt{RM}}\sum_{r=0}^{R-1}\sum_{k=0}^{RM-1}s_{r}\left[\tilde{k}\right]e^{j2\pi rp/R}e^{-j2\pi \tilde{m}k/RM}+\eta_{\tilde{m}}, \end{array}$$

where $k=pM+\tilde{k}$, $\tilde{k}\in \left[0,M-1\right]$, and p∈[0,R−1]. According to the definition of $s_{r}\left[k\right]$ in (1), the following equation holds,

(12) $$\begin{array}{c} s_{r}\left[k\right]=s_{r}\left[\tilde{k}\right],\;\;\;\;\;k=pM+\tilde{k}. \end{array}$$

As a result, substituting (1) into (11), Equation (11) can be rewritten as

(13) $$\begin{array}{cc} y_{\tilde{m}}=\frac{H_{\tilde{m}}}{M\sqrt{R}}\sum_{r=0}^{R-1}\sum_{k=0}^{RM-1}\sum_{m_{0}=0}^{M-1} & d_{m_{0},r}e^{j2\pi k(m_{0}R-\tilde{m}+r)/RM}\\ \; & \times e^{-j2\pi r\tilde{k}/RM}+\eta_{\tilde{m}}. \end{array}$$

Define $\tilde{m}=mR+p$, where m∈[0,M−1] and p∈[0,R−1]. Then, Equation (13) can be rewritten as

(14) $$\begin{array}{cc} \; & y_{mR+p}\\ \; & \;=\;\frac{H_{mR+p}}{M\sqrt{R}}\;\sum_{r=0}^{R-1}\;\sum_{k=0}^{RM-1}\;\sum_{m_{0}=0}^{M-1}\;d_{m_{0},r}e^{j2\pi k(m_{0}R+r-mR-p)/RM}\\ \; & \;\;\;\;\;\;\;\;\;\;\;\;\;\;\;\;\;\times e^{-j2\pi r\tilde{k}/RM}\;+\;\eta_{mR+p}. \end{array}$$

Note that the orthogonality condition of SR-OFDM always holds, as follows,

(15) $$\begin{array}{c} \sum_{k=0}^{RM-1}\;\;e^{j2\pi k(m_{0}R+r-mR-p)/RM}e^{-j2\pi r\tilde{k}/RM}=\left\{\begin{array}{c} \varepsilon ,\;\;\mathrm{if}\;p=r,\\ 0,\;\;\mathrm{otherwise}, \end{array}\right. \end{array}$$

where $k=pM+\tilde{k}$, $m_{0},\tilde{k}\in \left[0,M-1\right]$, p,r∈[0,R−1], and ε≠0. Note that ε is non-zero. According to (15), Equation (14) can be rewritten as

(16) $$\begin{array}{cc} y_{mR+r}=\frac{H_{mR+r}}{M\sqrt{R}}\;\;\sum_{m_{0}=0}^{M-1}\;\;d_{m_{0},r}\;\;\; & \sum_{k=0}^{RM-1}\;\;e^{j2\pi k(m_{0}-m)/M}\\ \; & \;\;\;\;\times e^{-j2\pi r\tilde{k}/RM}+\eta_{mR+r}. \end{array}$$

**Figure 3:**
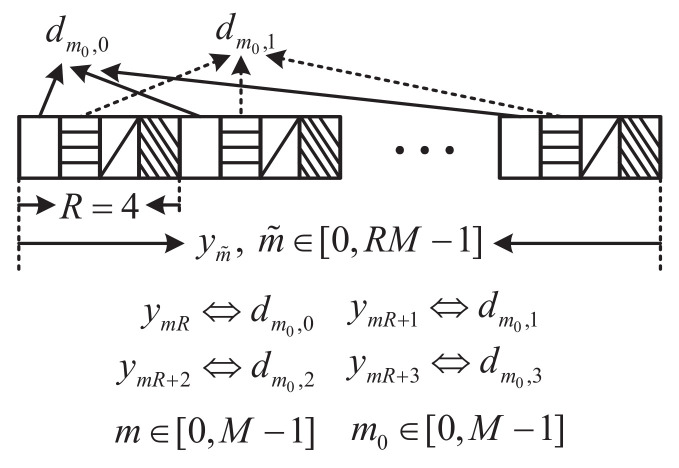
Separation of received signals of different $d_{m_{0},r}$ with respect to *r*, r∈[0,R−1], R=4.

Equation (16) indicates that, the signal $y_{\tilde{m}}$ with index $\tilde{m}=mR+r$ is determined only by $d_{m_{0},r}$ with respect to *r*, r∈[0,R−1]. In other words, the received signals of $d_{m_{0},r}$ and $d_{m_{0},p}$ can be separated in the time domain as presented in Figure 3. As a consequence, symbol demodulations of different $d_{m_{0},r}$ with respect to *r* can be conducted independently, lowering the receiver complexity. In the following, symbol demodulations of different $d_{m_{0},r}$ will be presented based on Equation (16).

#### 2.2.1. Symbol Demodulation of $d_{m,0}$

When r=0, Equation (16) can be rewritten as

(17) $$\begin{array}{cc} y_{mR} & =\frac{H_{mR}}{M\sqrt{R}}\;\;\sum_{m_{0}=0}^{M-1}\;\;d_{m_{0},0}\;\;\sum_{k=0}^{RM-1}\;\;e^{j2\pi k(m_{0}-m)/M}\;+\;\eta_{mR}. \end{array}$$

Note that the following equation holds,

(18) $$\begin{array}{c} \sum_{k=0}^{RM-1}e^{j2\pi k(m_{0}-m)/M}=\left\{\begin{array}{c} RM,\;\;\mathrm{if}\;m_{0}=m,\\ 0,\;\;\;\;\;\;\mathrm{otherwise}. \end{array}\right. \end{array}$$

Then, $y_{mR}$ can be obtained as

(19) $$\begin{array}{c} y_{mR}=\sqrt{R}H_{mR}d_{m,0}+\eta_{mR}, \end{array}$$

where m=0,1,…,M−1. When the channel frequency response $H_{mR}$ is known at the receiver, the single-tap equalization can be performed to recover $d_{m,0}$, i.e.,

(20) $$\begin{array}{cc} {\hat{d}}_{m,0} & =\frac{y_{mR}}{\sqrt{R}H_{mR}}, \end{array}$$

where m=0,1,…,M−1. It is worthwhile to point out that the factor $1/\sqrt{R}$ in (20) is a result of symbol repetition by *R* times in the SR-OFDM system.

#### 2.2.2. Symbol Demodulation of $d_{m,r},\;r\in \left[1,R-1\right]$

When r≠0, according to Equation (16), it can be obtained as

(21) $$\begin{array}{cc} y_{mR+r}=\frac{H_{mR+r}}{M\sqrt{R}}\;\;\sum_{m_{0}=0}^{M-1}\;\;d_{m_{0},r}\;\;\; & \sum_{k=0}^{RM-1}\;\;e^{j2\pi k(m_{0}-m)/M}\\ \; & \;\;\;\;\times e^{-j2\pi r\tilde{k}/RM}+\eta_{mR+r}, \end{array}$$

where $k=pM+\tilde{k},\;\tilde{k}\in \left[0,M-1\right],\;\mathrm{and}\;r\in \left[1,R-1\right]$. When $H_{mR}$ is known by the receiver, the single-tap equalizer can be applied, i.e.,

(22) $$\begin{array}{cc} {\tilde{y}}_{mR+r} & \;=\;\frac{y_{mR+r}}{H_{mR+r}}\;=\;\frac{1}{M\;\sqrt{R}}\;\;\sum_{m_{0}=0}^{M-1}\;\;d_{m_{0},r}\;\;\sum_{k=0}^{RM-1}\;\;\;e^{j2\pi k(m_{0}-m)/M}\\ \; & \;\;\;\;\;\;\;\;\;\;\;\;\;\;\;\;\;\;\times e^{-j2\pi r\tilde{k}/RM}+{\tilde{\eta }}_{mR+r}, \end{array}$$

where ${\tilde{\eta }}_{mR+r}$ is obtained by $\eta_{mR+r}/H_{mR+r}$.

From Equation (22), it is realized that the signals ${\tilde{y}}_{\tilde{m}R+r}$ are determined only by $d_{m_{0},r}$, and not interfered by $d_{m_{0},p}$ with p≠r. As a result, for the symbol recovery of $d_{m_{0},r}$, signals related to $d_{m_{0},r}$ are extracted, i.e.,

(23) $$\Upsilon_{\tilde{m},r}=\Phi \left(r\right)=\left\{\begin{array}{c} {\tilde{y}}_{mR+r},\;\;\;\mathrm{if}\;\tilde{m}=mR+r,\\ 0,\;\;\;\;\;\;\;\;\;\;\mathrm{otherwise}. \end{array}\right.$$

Subsequently, performing IDFT with length of RM on the signal $\Upsilon_{\tilde{m},r}$, it is obtained

(24) $$\begin{array}{cc} {\tilde{\Upsilon }}_{n,r} & \;=\;\frac{1}{\sqrt{R\;M}}\sum_{\tilde{m}=0}^{R\;M\;-\;1}\Upsilon_{\tilde{m},r}e^{j2\pi \tilde{m}n/RM}\\ \; & \;=\;\frac{1}{\sqrt{R\;M}}\sum_{\tilde{m}=0}^{R\;M\;-\;1}{\tilde{y}}_{mR+r}e^{j2\pi \tilde{m}n/RM}\\ \; & \;=\;\frac{1}{\sqrt{R^{2}\;M^{3}}}\;\;\;\sum_{\tilde{m}=0}^{R\;M\;-\;1}\;\;\sum_{m_{1}=0}^{M\;-\;1}\;\;d_{m_{1},r}\;\;\;\;\sum_{k=0}^{R\;M\;-\;1}\;\;\;e^{j(2\pi k\left(m_{1}-m\right)/M-2\pi r\tilde{k}/RM)}\\ \; & \;\;\;\;\;\;\;\;\;\;\;\;\;\;\;\;\;\;\;\;\times \;e^{j2\pi \tilde{m}n/RM}\;+\;{\bar{\eta }}_{n,r}, \end{array}$$

where $\tilde{m}=mR+r,\;\;\;r\in \left[1,R-1\right],\;\;\;k=pM+\tilde{k},\;\;\tilde{k}=0,1,\ldots ,M-1,\;\mathrm{and}\;m=0,1,\ldots ,M-1$. In addition, note that the noise term ${\bar{\eta }}_{n,r}$ in (24) is written as

(25) $$\begin{array}{c} {\bar{\eta }}_{n,r}=\frac{1}{\sqrt{RM}}\sum_{m=0}^{M-1}{\tilde{\eta }}_{mR+r}e^{-j2\pi (mR+r)n/RM}. \end{array}$$

Then, a phase coefficient is defined

(26) $$\begin{array}{cc} \Omega (r) & =e^{-j2\pi rp/R}=e^{-j2\pi r(n-\tilde{n})/RM}. \end{array}$$

**Figure 4:**
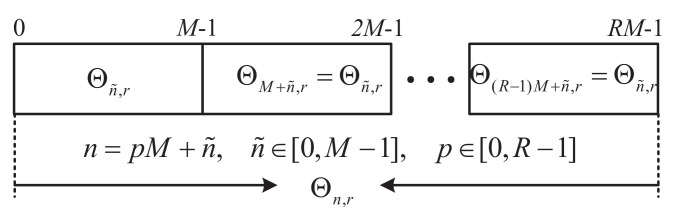
Repetition of signal $\Theta_{n,r}$.

Multiply ${\tilde{\Upsilon }}_{n,r}$ by Ω(r), $\Theta_{n,r}={\tilde{\Upsilon }}_{n,r}\Omega \left(r\right)$ is obtained as in (28), which is shown at the top of the next page, In addition, let $pM=n-\tilde{n}$ where n∈[0,RM−1] and $\tilde{n}\in \left[0,M-1\right]$, then, (28) is rewritten as in (29) (at the top of the next page). Then, it is obtained as

(27) $$\begin{array}{cc} \Theta_{n,r} & =\frac{1}{\sqrt{R^{2}M^{3}}}\sum_{m_{1}=0}^{M-1}d_{m_{1},r}e^{j2\pi nm_{1}/M}e^{j2\pi (nr-r\tilde{k}-rn+r\tilde{n})/RM}\\ \; & \;\;\;+{\hat{\eta }}_{n,r}\\ \; & =\frac{1}{\sqrt{M}}\sum_{m_{1}=0}^{M-1}d_{m_{1},r}e^{j2\pi nm_{1}/M}+{\hat{\eta }}_{n,r}, \end{array}$$

where $\tilde{m}=mR+r,\;\;r\in \left[1,R-1\right],\;\;n=pM+\tilde{n},\;\;k=pM+\tilde{k},\;\mathrm{and}\;\tilde{k}=0,1,\ldots ,M-1$. The noise term ${\hat{\eta }}_{n,r}$ is ${\bar{\eta }}_{n,r}e^{-j2\pi r(n-\tilde{n})/RM}$. From the analysis in (27), it is realized that for each *r*, the signal $\Theta_{n,r}$ has a structure of repetition, as shown in Figure 4, in which the noise term ${\hat{\eta }}_{n,r}$ is ignored.

(28) $$\begin{array}{cc} \Theta_{n,r} & =\frac{1}{\sqrt{R^{2}M^{3}}}\sum_{\tilde{m}=0}^{RM-1}\sum_{m_{1}=0}^{M-1}d_{m_{1},r}\sum_{k=0}^{RM-1}e^{j2\pi k(m_{1}-m)/M}e^{-j2\pi r\tilde{k}/RM}e^{j2\pi \tilde{m}n/RM}e^{-j2\pi rp/R}+{\hat{\eta }}_{n,r}\\ \; & =\frac{1}{\sqrt{R^{2}M^{3}}}\sum_{\tilde{m}=0}^{RM-1}\sum_{m_{1}=0}^{M-1}d_{m_{1},r}\sum_{k=0}^{RM-1}e^{j2\pi k(m_{1}-m)/M}e^{-j2\pi r\tilde{k}/RM}e^{j2\pi \tilde{m}n/RM}e^{-j2\pi rpM/RM}+{\hat{\eta }}_{n,r}. \end{array}$$

(29) $$\begin{array}{cc} \Theta_{n,r} & \;=\;\frac{1}{\sqrt{R^{2}M^{3}}}\;\;\sum_{\tilde{m}=0}^{RM-1}\;\sum_{m_{1}=0}^{M-1}\;\;d_{m_{1},r}\;\;\sum_{k=0}^{RM-1}\;\;e^{j2\pi k(m_{1}-m)R/RM}e^{-j2\pi r\tilde{k}/RM}e^{j2\pi \tilde{m}n/RM}e^{-j2\pi rn/RM}e^{j2\pi r\tilde{n}/RM}+{\hat{\eta }}_{n,r}\\ \; & \;=\;\frac{1}{\sqrt{R^{2}M^{3}}}\;\;\sum_{m_{1}=0}^{M-1}\;\;d_{m_{1},r}\;\;\sum_{k=0}^{RM-1}\;\sum_{\tilde{m}=0}^{RM-1}\;\;e^{-j2\pi \left(k-n\right)\tilde{m}/RM}e^{j2\pi kr/RM}e^{j2\pi km_{1}R/RM}e^{-j2\pi r\tilde{k}/RM}e^{-j2\pi rn/RM}e^{j2\pi r\tilde{n}/RM}\;+\;{\hat{\eta }}_{n,r}. \end{array}$$

Then, symbol demodulation is achieved by performing a RM-point DFT, i.e.,

(30) $$\begin{array}{cc} {\hat{\Theta }}_{\tilde{m},r} & =\frac{1}{\sqrt{RM}}\sum_{n=0}^{RM-1}\Theta_{n,r}e^{-j2\pi n\tilde{m}/RM}\\ \; & =\frac{1}{\sqrt{RM^{2}}}\sum_{n=0}^{RM-1}\sum_{\tilde{m}=0}^{M-1}d_{m,r}e^{j2\pi n(mR-\tilde{m})/RM}+{\overset{´}{\eta }}_{\tilde{m},r}\\ \; & =\left\{\begin{array}{cc} \sqrt{R}d_{m,r}+{\overset{´}{\eta }}_{\tilde{m},r},\;\;\; & \mathrm{if}\;\tilde{m}=mR,\\ {\overset{´}{\eta }}_{\tilde{m},r},\;\;\; & \mathrm{otherwise}, \end{array}\right. \end{array}$$

where

(31) $$\begin{array}{c} {\overset{´}{\eta }}_{\tilde{m},r}=\frac{1}{\sqrt{RM}}\sum_{n=0}^{RM-1}{\hat{\eta }}_{n,r}e^{-j2\pi n\tilde{m}/RM}. \end{array}$$

Finally, it is obtained as

(32) $$\begin{array}{cc} {\hat{d}}_{m,r} & =\frac{{\hat{\Theta }}_{mR,r}}{\sqrt{R}}=d_{m,r}+\frac{{\overset{´}{\eta }}_{mR,r}}{\sqrt{R}}, \end{array}$$

where r∈[1,R−1]. Similar to the classical OFDM, SR-OFDM can overcome the multi-path effect with the help of CP. Note that CP is inserted for each OFDM symbol, while CP is shared by one block consisting of several symbols in the proposed SR-OFDM. As a result, compared to OFDM, the overhead of CP is lowered significantly in SR-OFDM. In addition, it is obvious that the symbol demodulation of SR-OFDM is different from that in OFDM, resulting in more complex signal processing for its combination with the multiple input multiple output (MIMO) technique [20,21].

## 3. SR-OFDM under a Timing Synchronization Error

**Figure 5:**
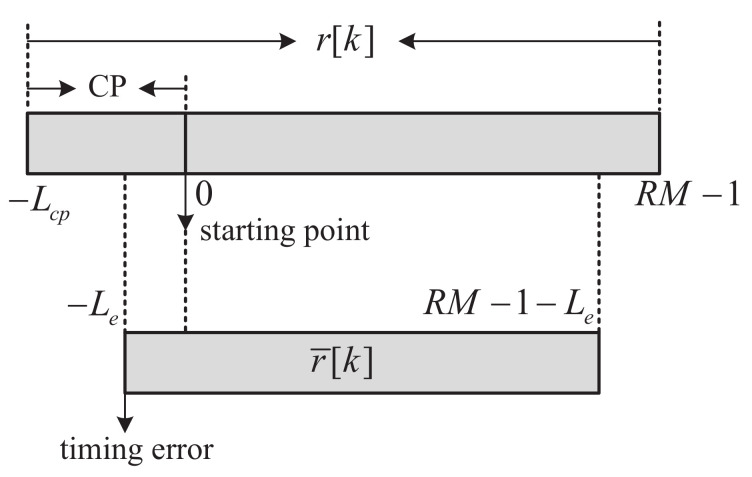
Received signal under a timing error.

For the symbol demodulation, the starting position of one symbol is required to be obtained from the received signal, which is based on the timing synchronization. However, due to the noise or interference, denoted as $L_{e}$ as shown in Figure 5, degrading the performance of the symbol demodulation. In this section, we focus on the performance of SR-OFDM under a timing error. It will be proven that when the timing error is within the CP, symbol demodulation with a single-tap equalizer can be performed without causing any inter-symbol interference as long as the CP length is large enough. It will also demonstrate that under such a condition, the property that the signals of different data symbols are separated in the time domain still remains, which ensures a low complexity of symbol demodulation.

### 3.1. Received Signal with Timing Synchronization Error

When the time synchronization is perfect, the signal after the CP removing, $\tilde{r}\left[k\right],$k=0,1,…,RM−1, is obtained as in Equation (6). Under a time synchronization error, denoted as $L_{e}$, the signal can be obtained as

(33) $$\begin{array}{cc} \bar{r}\left[k\right] & =\sum_{l=0}^{L_{h}-1}h\left[l\right]\tilde{s}\left[k-l\right]+\eta \left[k\right], \end{array}$$

where $k=-L_{e},-L_{e}+1,\ldots ,RM-1-L_{e}$. Note that it is assumed that the time synchronization error is within the CP, and the value of $L_{cp}-L_{e}$ is still larger than the maximum channel delay. Otherwise, the inter-symbol interference will occur definitely, and this will go beyond our concern.

Subsequently, the RM-point DFT is performed on $\bar{r}\left[k\right]$, and since the indexes of $\bar{r}\left[k\right]$ are $-L_{e},-L_{e}+1,\ldots ,RM-1-L_{e}$, the result after the DFT can be written as

(34) $$\begin{array}{c} {\bar{y}}_{\tilde{m}}=\frac{1}{\sqrt{RM}}\sum_{k=0}^{RM-1}\bar{r}\left[k-L_{e}\right]e^{-j2\pi \tilde{m}k/RM}, \end{array}$$

where $\tilde{m}=0,1,\ldots ,RM-1$. Substituting (4) and (33) into (34), it is obtained as

(35) $$\begin{array}{cc} {\bar{y}}_{\tilde{m}} & \;=\;\frac{1}{\sqrt{R\;M}}\;\;\sum_{k=0}^{R\;M\;-\;1}\sum_{l=0}^{L_{h}\;-\;1}\;\;h\left[l\right]\tilde{s}\left[k-l-L_{e}\right]e^{-j2\pi \tilde{m}k/R\;M}\;+\;\eta_{\tilde{m}}\\ \; & \;=\;\frac{1}{\sqrt{R\;M}}\;\;\sum_{k=0}^{R\;M\;-\;1}\sum_{r=0}^{R-1}\sum_{l=0}^{L_{h}\;-\;1}\;\;h\left[l\right]{\tilde{s}}_{r}\left[k-l-L_{e}\right]e^{-j2\pi \tilde{m}k/R\;M}\;+\;\eta_{\tilde{m}}\\ \; & \;=\;\frac{1}{\sqrt{R\;M}}\;\;\sum_{k=-l-L_{e}}^{R\;M\;-\;1\;-\;l\;-\;L_{e}}\sum_{r=0}^{R\;-\;1}\;\sum_{l=0}^{L_{h}\;-\;1}\;\;h\left[l\right]{\tilde{s}}_{r}\left[k\right]e^{-j2\pi \tilde{m}\left(k+l+L_{e}\right)/RM}\;+\;\eta_{\tilde{m}}\\ \; & \;=\;\frac{1}{\sqrt{R\;M}}\;\;\sum_{r=0}^{R\;-\;1}\;\;\left[\;\sum_{l=0}^{L_{h}\;-\;1}\;\;h\left[l\right]e^{-j2\pi \tilde{m}\left(l\;+\;L_{e}\right)\;/\;R\;M}\;\right]\;\;\sum_{k=-\;l\;-\;L_{e}}^{R\;M\;-\;1\;-\;l\;-\;L_{e}}\;\;\;\;\;\;\;\;\;{\tilde{s}}_{r}\left[k\right]e^{-j2\pi \tilde{m}k\;/\;R\;M}\\ \; & \;\;\;+\eta_{\tilde{m}}. \end{array}$$

Due to the fact that ${\tilde{s}}_{r}\left[k\right]$ is a signal with the circular structure according to (3), Equation (35) can be rewritten as

(36) $$\begin{array}{cc} {\bar{y}}_{\tilde{m}} & \;=\;\frac{1}{\sqrt{R\;M}}\;\;\sum_{r=0}^{R-1}\;\;\left[\;\sum_{l=0}^{L_{h}\;-\;1}\;\;h\left[l\right]e^{-\;j2\pi \tilde{m}\left(\;l\;+\;L_{e}\;\right)\;/\;R\;M}\;\right]\;\;\sum_{k=0}^{R\;M\;-\;1}\;\;\;{\tilde{s}}_{r}\left[k\right]e^{-\;j2\pi \tilde{m}k\;/\;R\;M}\\ \; & \;\;\;+\eta_{\tilde{m}}\\ \; & \;=\;\frac{e^{-j2\pi \tilde{m}\;L_{e}\;/\;R\;M}\;H_{\tilde{m}}}{\sqrt{RM}}\;\sum_{r=0}^{R\;-\;1}\;\;\sum_{k=0}^{R\;M\;-\;1}\;\;\;{\tilde{s}}_{r}\left[k\right]e^{-j2\pi \tilde{m}k/RM}\;+\;\eta_{\tilde{m}}. \end{array}$$

Note that the term in (36), $\frac{e^{-j2\pi \tilde{m}L_{e}/RM}H_{\tilde{m}}}{\sqrt{RM}}$ only depends on the parameter $\tilde{m}$ for a fixed timing synchronization error $L_{e}$. Then, similar to derivations from (11) to (16), Equation (36) can be rewritten as

(37) $$\begin{array}{cc} {\bar{y}}_{mR+r} & =\frac{H_{mR+r}}{M\;\sqrt{R}}\;\sum_{m_{0}=0}^{M\;-\;1}\;\;\sum_{k=0}^{R\;M\;-\;1}\;\;d_{m_{0},r}e^{j2\pi k(m_{0}-m)\;/\;M}e^{-j2\pi r\tilde{k}\;/\;R\;M}\\ \; & \;\times e^{-j2\pi \left(mR+r\right)L_{e}/RM}+\eta_{mR+r}. \end{array}$$

Equation (37) indicates that for a fixed timing synchronization error $L_{e}$, the signal ${\bar{y}}_{mR+r}$ with index $\tilde{m}=mR+r$ is determined only by $d_{m_{0},r}$ with respect to *r*, r∈[0,R−1]. In other words, for $d_{m_{0},r}$ with different *r*, their received signals are still separated, exactly as the conclusion presented in (16). The separation of received signals makes it possible to perform symbol demodulation independently for each *r*, ensuing a low complexity of the receiver. It is easily observed that when the term $e^{-j2\pi \left(mR+r\right)L_{e}/RM}H_{mR+r}$ in (37) can be removed, the symbol demodulation can be performed according to Equations (17)–(32) (within Section 2.2.1) without any interference among symbols.

Due to the reason that the timing synchronization error $L_{e}$ is unknown, the key to symbol demodulation is the elimination of $L_{e}$. In the following subsection, methods for symbol demodulation are proposed under the timing synchronization error $L_{e}$.

### 3.2. Symbol Demodulation under Timing Synchronization Error

According to (37), the estimations of channel frequency responses and the timing synchronization error are required before the symbol demodulation. In wireless communications, the channel estimation is usually conducted by using pilots [22,23], i.e., the transmitter sends a reference signal that has been known by the receiver in advance. This method of pilots can also be used for the estimations of channel frequency responses and the timing synchronization error in Equation (37).

For the branch of r=0, similar to the derivations of (17)–(19), ${\bar{y}}_{mR+r}$ in (37) can be rewritten as

(38) $$\begin{array}{cc} {\bar{y}}_{mR} & =\sqrt{R}e^{-j2\pi mRL_{e}/RM}H_{mR}d_{m,0}+\eta_{mR}, \end{array}$$

where m=0,1,…,M−1. From (38), the received signal of the branch r=0 in SR-OFDM is the product of the channel frequency response $H_{mR}$ and transmitted symbol $d_{m,0}$, which is similar to the classical OFDM. Consequently, for the proposed SR-OFDM, the branch of r=0 preserves the simplicity of the classical OFDM, which is helpful for the pilot design and channel estimation.

According to (38), the term $e^{-j2\pi mRL_{e}/RM}H_{mR}$ can be easily estimated by making $d_{m,0}$ as pilots, and the estimation can be written as

(39) $$\begin{array}{cc} {\hat{H}}_{mR} & \;=\;\frac{{\bar{y}}_{mR}}{\sqrt{R}d_{m,0}}\;=\;e^{-j2\pi mRL_{e}/RM}H_{mR}\;+\;\frac{\eta_{mR}}{\sqrt{R}d_{m,0}}, \end{array}$$

where m=0,1,…,M−1. When the noise term $\frac{\eta_{mR}}{\sqrt{R}d_{m,0}}$ in (39) is removed, ${\hat{H}}_{mR}$ is the product of $e^{-j2\pi mRL_{e}/RM}$ and $d_{m,0}$. Subsequently, the single-tap equalization can be performed on ${\bar{y}}_{mR}$ to remove the effects of the channel and timing synchronization error, and transmitted symbols $d_{m,r}$ for the branch r=0 can be recovered perfectly.

**Figure 6:**
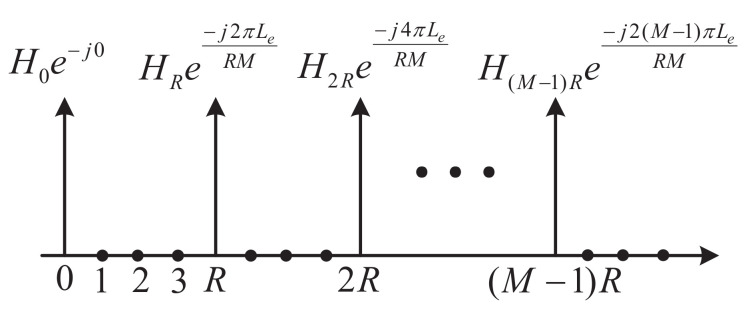
Estimation of the channel and timing synchronization error by using branch r=0 in SR-OFDM, taking R=4 as an example.

It should be noted that the proposed SR-OFDM consists of *R* branches of independent data streams, as shown in Figure 1. To recover all branches of transmitted symbols, the estimations of $e^{-j2\pi \tilde{m}L_{e}/RM}H_{\tilde{m}}$ are required for each $\tilde{m}\in \left\{0,1,\ldots ,RM-1\right\}$. However, Equation (39) only provides the estimations of $e^{-j2\pi mRL_{e}/RM}H_{mR}$ for m={0,1,…,M−1}, as shown in Figure 6 where *R* is set to 4 as an example. Therefore, algorithms are required to obtain the the whole estimations of $e^{-j2\pi \tilde{m}L_{e}/RM}H_{\tilde{m}}$ only by the branch r=0 in SR-OFDM. As a remark, the estimations of $e^{-j2\pi \tilde{m}L_{e}/RM}H_{\tilde{m}}$ can be also achieved by other branches r∈[1,R−1] instead of only the branch r=0. For the sake of simplicity, this paper performs the estimations of $e^{-j2\pi \tilde{m}L_{e}/RM}H_{\tilde{m}}$ only by using pilots in the branch r=0.

**Figure 7:**
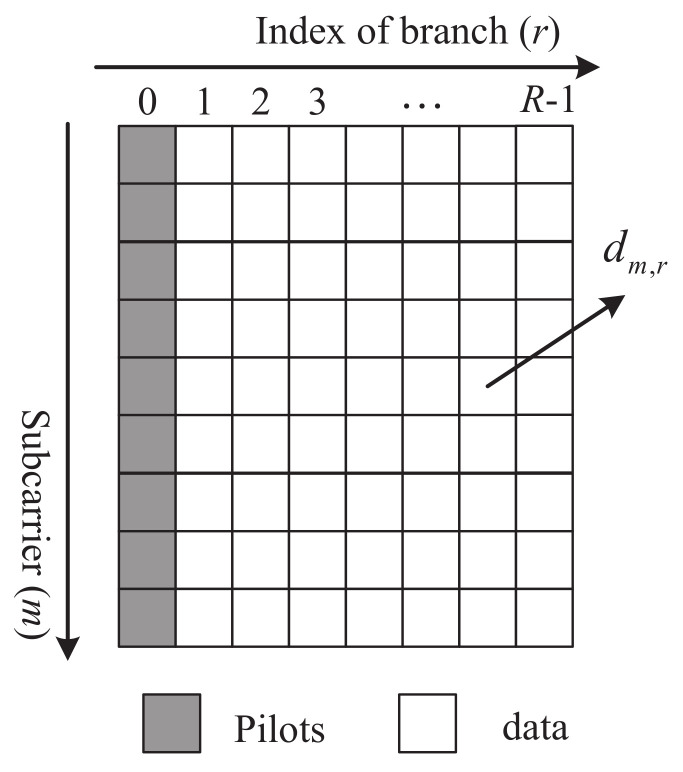
Pilot design in the proposed SR-OFDM.

As shown in Figure 7, $d_{m,0}$ are set to the pilot symbols, and $d_{m,r},\;r\neq 0$ are data symbols. Note that the variable *r* in Figure 7 is the index of branch for $d_{m,r}$, not the index of the symbol in the time domain. Then, according to (39), the joint estimation of the frequency channels and the timing synchronization error can be obtained for the indices 0,R,…,(M−1)R, i.e.,

(40) $$\begin{array}{cc} {\hat{H}}_{mR} & =\frac{{\bar{y}}_{mR}}{\sqrt{R}d_{m,0}}, \end{array}$$

where m=0,1,…,M−1. In the following, according to the estimations ${\hat{H}}_{mR},\;m=0,$1,…,M−1, two interpolation algorithms are presented to obtain the whole estimations $e^{-j2\pi \tilde{m}L_{e}/RM}H_{\tilde{m}},\;\tilde{m}=0,1,\ldots ,RM-1$.

#### 3.2.1. DFT-Based Estimation

First, the frequency–domain estimations in (40) can be converted into the time–domain estimations by the *M*-point IDFT operation, i.e.,

(41) $$\begin{array}{c} \hat{h}\left[k\right]=\sum_{m=0}^{M-1}{\hat{H}}_{mR}e^{j2\pi mk/M}, \end{array}$$

where k=0,1,…,M−1. Substituting (39) into (41), it is obtained,

(42) $$\begin{array}{cc} \hat{h}\left[k\right] & =\sum_{m=0}^{M-1}\left(e^{-j2\pi mRL_{e}/RM}H_{mR}+\frac{\eta_{mR}}{\sqrt{R}d_{m,0}}\right)e^{j2\pi mk/M}\\ \; & =\sum_{m=0}^{M-1}H_{mR}e^{-j2\pi mRL_{e}/RM}e^{j2\pi mk/M}+\xi_{k}\\ \; & =\sum_{m=0}^{M-1}\sum_{l=0}^{L_{h}-1}h\left[l\right]e^{-j2\pi mRl/RM}e^{-j2\pi mRL_{e}/RM}e^{j2\pi mk/M}\\ \; & \;\;\;\;+\xi_{k}\\ \; & =h\left[k-L_{e}\right]+\xi_{k}, \end{array}$$

where k=0,1,…,M−1 and $\xi_{k}=\sum_{m=0}^{M-1}\frac{\eta_{mR}}{\sqrt{R}d_{m,0}}e^{j2\pi mk/M}$ satisfies the Gaussian distribution. Note that h[k] is the multi-path fading channel with the maximum channel delay $L_{h}<M$. After obtaining the maximum channel delay, a time–domain window g[k] can be designed to extract the non-zero channel coefficients,

(43) $$\begin{array}{c} \begin{array}{c} g\left[k\right]=\left\{\begin{array}{cc} 1, & 0\leq k<L_{h}+L_{e},\\ 0, & L_{h}+L_{e}\leq k<M, \end{array}\right. \end{array} \end{array}$$

Then, it is written as

(44) $$\begin{array}{c} \bar{h}\left[k\right]=g\left[k\right]\hat{h}\left[k\right], \end{array}$$

where k=0,1,…,M−1. Moreover, the function g[k] is helpful to improve the estimation performance since it gets rid of the noise at $k=L_{h}+L_{e},L_{h}+L_{e}+1,\ldots ,M-1$.

After that, the frequency interpolations can be obtained by an RM-point DFT operation, i.e.,

(45) $$\begin{array}{cc} {\hat{H}}_{\tilde{m}} & =\sum_{k=0}^{R\;M-\;1}\bar{h}\left[k\right]e^{-j2\pi \tilde{m}k/RM}=e^{-j2\pi \tilde{m}L_{e}/RM}H_{\tilde{m}}+{\bar{\xi }}_{k}, \end{array}$$

where $\tilde{m}=0,1,\ldots ,RM-1$ and ${\bar{\xi }}_{k}$ is the noise term, which can be obtained by $\sum_{k=0}^{RM-1}g\left[k\right]\xi_{k}e^{-j2\pi \tilde{m}k/RM}$.

On the basis, a single-tap equalizer, i.e., $1/{\hat{H}}_{\tilde{m}}$, can be performed on the received signal ${\bar{y}}_{mR+r}$ in (37). When ignoring the noise for simplicity, it is obtained as

(46) $$\begin{array}{cc} {\bar{y}}_{mR+r} & \;=\;\frac{1}{M\;\sqrt{R}}\;\sum_{m_{0}=0}^{M-1}\;\sum_{k=0}^{RM-1}\;\;\;\;d_{m_{0},r}e^{j2\pi k(m_{0}-m)/M}e^{-j2\pi r\tilde{k}/RM}, \end{array}$$

where $\tilde{m}=mR+r$ with m∈[0,M−1] and r∈[1,R−1]. Then, the transmitted symbols $d_{m,r}$ with [1,R−1] can be recovered perfectly similar to Equations (22)–(32). Note that the symbol demodulation of $d_{m,r}$ with r=0 can be directly obtained according to (38).

#### 3.2.2. Linear Interpolation Estimation

According to the analysis in (43) and (44), $e^{-j2\pi \tilde{m}L_{e}/RM}H_{\tilde{m}}$ corresponds to the time–domain channel that suffered from a delay, i.e., an updated time–domain channel $h[k-L_{e}]$. Therefore, the estimations ${\hat{H}}_{mR},\;m=0,1,\ldots ,M-1$ in (40) are the frequency responses of $h[k-L_{e}]$ at the frequencies 0,R,…,(M−1)R. Suppose that the channel frequency responses vary slowly at several frequency points. The simple linear interpolation method can be applied to obtain the estimations of $e^{-j2\pi \tilde{m}L_{e}/RM}H_{\tilde{m}},\;\tilde{m}=0,1,\ldots ,RM-1$, i.e.,

(47) $$\begin{array}{c} {\hat{H}}_{mR+r}=\frac{r}{R}{\hat{H}}_{mR}+\frac{R-r}{R}{\hat{H}}_{\left(m+1\right)R}, \end{array}$$

where *m* is the subcarrier index, and r=1,2,…,R−1. Similar to (46), the single-tap equalizer, i.e., $1/{\hat{H}}_{\tilde{m}}$, can be performed, and symbol demodulation can be obtained.

The linear interpolation estimation can be implemented easily due to the low complexity. However, under a channel with strong frequency selectivity, the linear interpolation estimation in (47) will suffer from a little distortion, which may result in a degraded performance for high-order modulation or high signal-to-noise (SNR). Compared with the linear interpolation estimation, the DFT-based method can achieve the accurate estimation of $e^{-j2\pi \tilde{m}L_{e}/RM}H_{\tilde{m}}$. Furthermore, the design of the time–domain window in (43) can significantly improve the estimation performance since it removes much noise. In spite of this, the DFT-based method requires the maximum channel delay $L_{h}$ as the a priori knowledge.

## 4. Performance Analysis

### 4.1. Spectral Efficiency

In this subsection, we analyze the spectral efficiency of SR-OFDM under an AWGN channel and show the advantage compared to the classical OFDM. According to the Shannon theory [24,25], the spectral efficiency depends on the received SNR and the time used for the useful signals.

First, the SNR of symbol demodulation is analyzed. For simplicity, assume that the transmit power of for each symbol $d_{m,r}$ is 1, and the variance of the channel noise η[k] is $\sigma^{2}$. According to Equations (20) and (32), SNR of symbol demodulation can be easily obtained as

(48) $$\begin{array}{c} {\mathrm{SNR}}_{o}=\frac{R}{\sigma^{2}}. \end{array}$$

The factor *R* in (48) is due to the symbol repetition in the proposed SR-OFDM. For a fair transmit power for each symbol, the SR-OFDM and OFDM exhibit the same received SNR of symbol demodulation.

As for the transmission time, according to Section 2.1, one symbol $d_{m,r}$ is repeated for *R* times, and therefore, it costs the time RT when the CP overhead is not considered. Due to the design of *R* branches as shown in Figure 1, the proposed SR-OFDM can transmit RM data symbols totally during the time RT (ignoring the CP overhead), where *M* is the number of subcarriers. It is well known that the classical OFDM can also transmit RM data symbols totally during the time RT [26] (ignoring the CP overhead). Therefore, when ignoring the CP overhead and ensuring the same transmit power for fairness, the proposed SR-OFDM will have the same spectral efficiency as the classical OFDM. When CP is considered, the overhead of the classical OFDM is $\frac{T_{cp}}{T+T_{cp}}$ since CP is required for each symbol, whereas the overhead of the proposed SR-OFDM is $\frac{T_{cp}}{RT+T_{cp}}$, where $T_{cp}$ is the time duration of CP.

**Figure 8:**
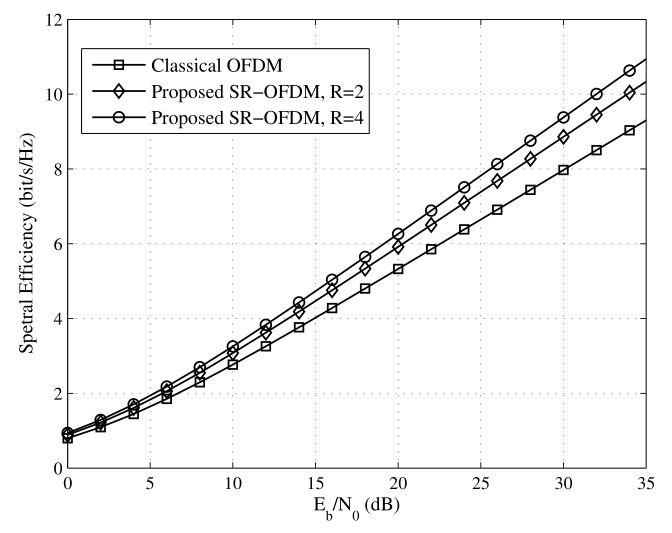
Spectral efficiency comparison between the SR-OFDM and OFDM under an AWGN channel.

As shown in Figure 8, the spectral efficiency of the proposed SR-OFDM is compared with the classical OFDM, in which $T_{cp}$ is set to 0.25 T exactly as IEEE 802.11a [8]. The proposed SR-OFDM offers a significant spectral efficiency gain over the classical OFDM, especially for a large *R*, thanks to the reduction of CP in the proposed SR-OFDM.

### 4.2. Complexity Comparison

In this subsection, we compare the complexity of the proposed SR-OFDM with the classical OFDM. In general, the overall number of complex-valued multiplications can be used to evaluate the complexity [27].

As presented in Section 2.1, the complexity of the SR-OFDM transmitter mainly stems from *R* branches of IDFT transforms and the multiplications with the phase coefficients $e^{j2\pi rp/R}$. Note that the *M*-point IDFT can be implemented by the *M*-point inverse fast Fourier transform (IFFT) that has a multiplication number of $M\log_{2}\left(M\right)$. Therefore, the complexity of the SR-OFDM transmitter is

(49) $$\begin{array}{c} R\left(M\log_{2}\left(M\right)+RM\right)=RM\log_{2}\left(M\right)+R^{2}M, \end{array}$$

whereas the complexity of the transmitter in the classical OFDM is $RM\log_{2}\left(M\right)$ for *R* symbols.

As for the receiver, although the first branch of SR-OFDM, i.e., $d_{m,0}$, preserves the simplicity of OFDM and only the DFT transform is required, the rest of the R−1 branches have a higher complexity than the classical OFDM. As presented in Section 2.2, the complexity of the SR-OFDM receiver mainly stems from an RM-point DFT, R−1 branches of IDFT, the multiplications with phase coefficients, and DFT. As a result, the complexity of the SR-OFDM receiver is

(50) $$\begin{array}{cc} \; & \;\;\;\;RM\log_{2}\left(RM\right)+2\left(R-1\right)RM\log_{2}\left(RM\right)+\left(R-1\right)RM\\ \; & =\left(2R-1\right)RM\log_{2}\left(RM\right)+\left(R-1\right)RM. \end{array}$$

As a comparison, the complexity of the classical OFDM receiver is $RM\log_{2}\left(M\right)$ for *R* symbols.

According to Table 2, the complexity of the proposed SR-OFDM is higher than the classical OFDM. However, it is noted that the increase of complexity mainly depends on the parameter *R*, which is limited by the channel coherence time. Therefore, a small *R* makes the complexity of SR-OFDM acceptable in practice. For instance, M=2048 and R=2, the complexity ratio of the SR-OFDM and OFDM receivers is

(51) $$\begin{array}{c} \frac{\left(2R-1\right)RM\log_{2}\left(RM\right)+\left(R-1\right)RM}{RM\log_{2}\left(M\right)}\approx 3.3636,\;\;R=2. \end{array}$$

For the parameters M=2048 and R=4, the complexity ratio of the SR-OFDM and OFDM receivers is

(52) $$\begin{array}{c} \frac{\left(2R-1\right)RM\log_{2}\left(RM\right)+\left(R-1\right)RM}{RM\log_{2}\left(M\right)}\approx 8.5455,\;\;R=4. \end{array}$$

### 4.3. PAPR Comparison

**Figure 9:**
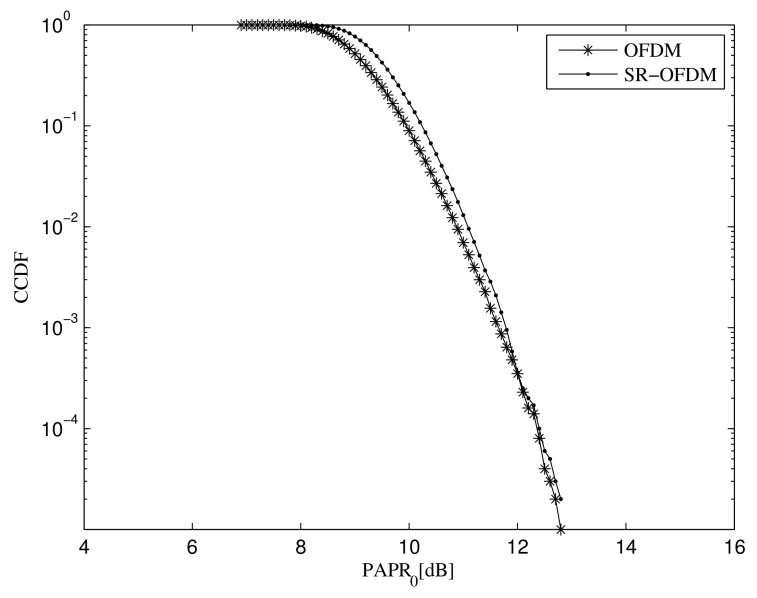
PAPR comparison between the classical OFDM and the proposed SR-OFDM.

As it is well known, PAPR in multi-carrier modulations is mainly caused by the superposition of all subcarrier signals [28]. However, the signal superposition in the proposed SR-OFDM is not only from the subcarriers but also the branches as shown in Figure 1. To evaluate the PAPR, Figure 9 shows the complementary cumulative distribution function (CCDF) curves of the transmitted signals in SR-OFDM and OFDM, in which the number of subcarriers is M=2048. From the simulation results, we see that the PAPR difference of SR-OFDM and OFDM is very limited. The reason is as follows. It has been demonstrated in [28] that the transmitted signal of the classical OFDM follows the Gaussian distribution when the subcarrier number is large enough and data symbols are independent and identically distributed (i.i.d). According to the principle diagram in Figure 1, the transmitted signal of the proposed SR-OFDM is equivalent to the superposition of multiple OFDM signals, which also approaches the Gaussian distribution and leads to a similar PAPR with the classical OFDM.

## 5. Simulation Results

In this section, bit error rate (BER) and mean square error (MSE) are carried out to validate the performance of the proposed SR-OFDM under multi-path fading channels and a time synchronization error. In simulations, we consider the SR-OFDM system with R=2 and subcarrier number M=2048, in which quadrature phase shift keying (QPSK) is adopted. The subcarrier spacing is set to 15,000 Hz exactly as the long-term evolution (LTE) standard [29], and then the duration one symbol will be $T=\frac{1}{15000}s=66.7\;\mu$s. The time duration of CP is set to 0.25 T, which is the same as the length of extended CP in LTE [29]. Moreover, the multi-path channels are adopted by considering the Stanford University interim (SUI) models [30], and the detailed channel profiles are shown in Table 3 [30], in which the channel SUI-5 has a larger path delay and exhibits a stronger frequency selectivity than the SUI-3 channel.

**Figure 10:**
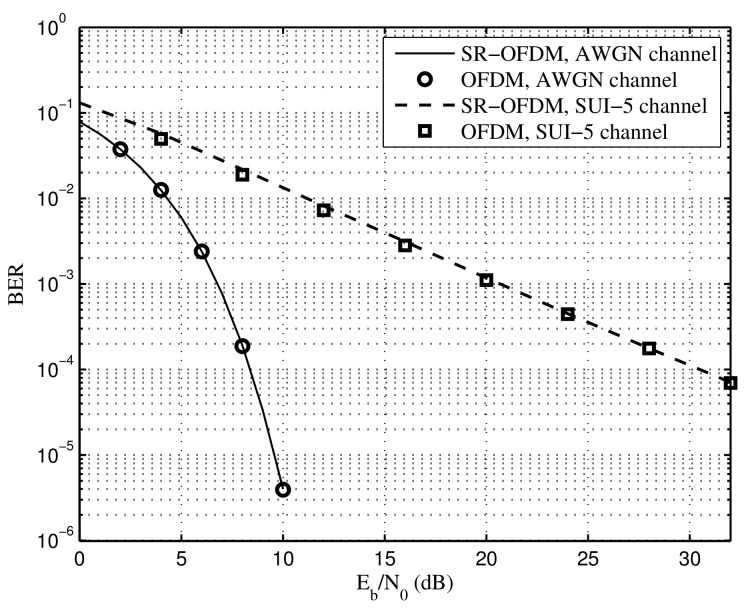
BER comparison between the classical OFDM and the proposed SR-OFDM, perfect channel estimation.

Figure 10 depicts the BER comparison between the classical OFDM and the proposed SR-OFDM under the AWGN channel and the SUI-5 channel, respectively, in which the perfect channel estimation and time synchronization are assumed. For a fair comparison, the total transmit power of the proposed SR-OFDM is the same as that of the classical OFDM when ignoring the CP overhead. Simulation results show that the proposed SR-OFDM achieves the same BER performance as the classical OFDM, which indicates that the multi-path channel can be overcome without causing the interference among symbols. Note that exactly as the classical OFDM, the simple single-tap equalization still works in the proposed SR-OFDM as the equations in (20) and (22). Furthermore, it is demonstrated that the received signals of different $d_{m,r}$ with respect to *r* are separated after the DFT module at the receiver, as in (16), reducing the complexity of symbol demodulation in the proposed SR-OFDM. As a result, the proposed SR-OFDM is an efficient multi-carrier waveform, which reduces the CP overhead significantly.

**Figure 11:**
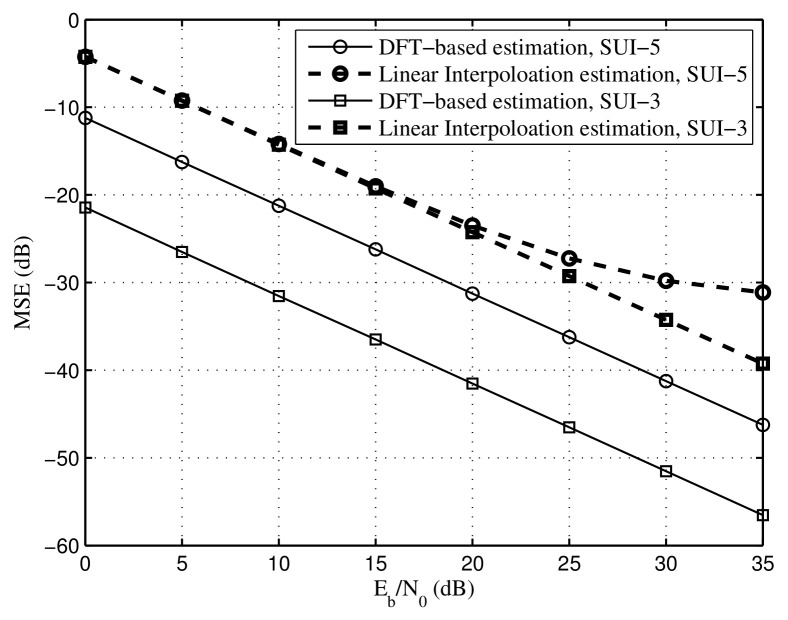
MSE of the proposed DFT-based and linear interpolation methods, time synchronization error $L_{e}=0.9\;\mu$s.

When a time synchronization error exists for the proposed SR-OFDM, it is demonstrated that the time synchronization error and the multi-path channel can be jointly overcome by a single-tap equalizer, as the equation in (37). The joint estimation of time synchronization error and the multi-path channel can be achieved by the proposed algorithms, i.e., the DFT-based estimation and the linear interpolation estimation in Section 3.2. Figure 11 depicts the MSE performances of the proposed algorithms, in which the time synchronization error is set to 0.9μs. Note that the length of CP is 0.25 T ≈16.7μs. The maximum channel delay of SUI-3 is 0.9μs, and the maximum channel delay of SUI-5 is 10μs. As a result, the time synchronization error 0.9μs is within the CP, and the CP length subtracting the time synchronization error is still larger than the maximum channel delay for both SUI-3 and SUI-5, i.e., 16.7μs − 0.9 μs > 10 μs > 0.9 μs. From the simulation results, it is observed that the proposed DFT-based estimation achieves a better MSE performance than the linear interpolation estimation thanks to the time–domain window in the proposed DFT-based estimation. It is worthwhile to point out that the priori knowledge of maximum channel delay is required in the proposed DFT-based estimation. Moreover, for the proposed DFT-based estimation, the MSE of SUI-3 is lower than that of SUI-5, which is because a shorter time–domain window is required, and more noise is removed for the SUI-3 channel. Furthermore, for the proposed linear interpolation estimation, there exists a performance floor of MSE under the channel SUI-5 at the high SNR region compared with the channel SUI-3, which is due to the strong frequency selectivity in the SUI-5 channel.

**Figure 12:**
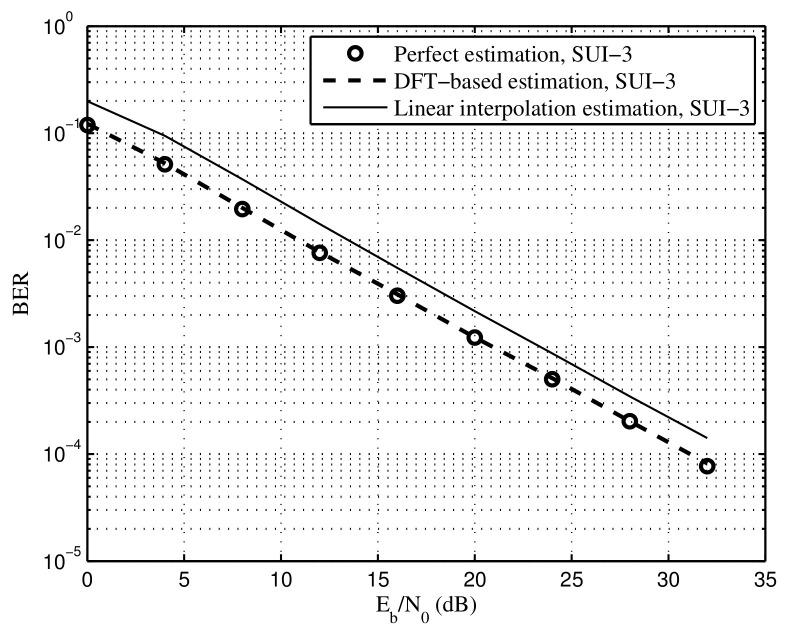
BER of the proposed DFT-based and linear interpolation methods under the SUI-3 channel, time synchronization error $L_{e}=0.9\;\mu$s.

**Figure 13:**
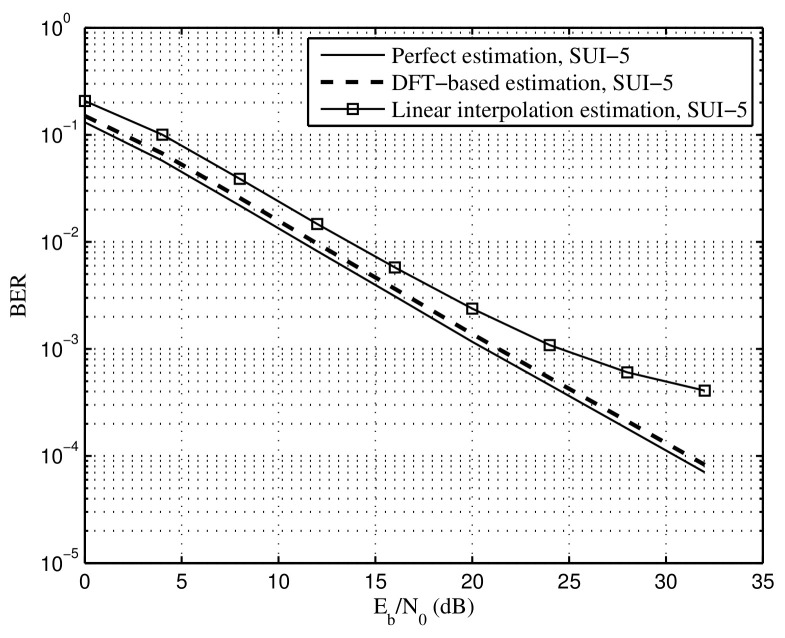
BER of the proposed DFT-based and linear interpolation methods under the SUI-5 channel, time synchronization error $L_{e}=0.9\;\mu$s.

Figure 12 and Figure 13 show BER of the proposed DFT-based and linear interpolation methods under SUI-3 and SUI-5, respectively, in which the time synchronization error is set to $L_{e}=0.9\;\mu$s. For both SUI-3 and SUI-5, the BER of the DFT-based method approaches the performance of the perfect estimation, indicating that most of the noise is removed in the proposed DFT-based estimation. Note that the priori knowledge of maximum channel delay is required in the proposed DFT-based method. Moreover, the proposed linear interpolation method suffers from a performance floor of BER at high SNR under the SUI-5 channel, which means that a bit distortion for the estimation of the channel exists as well as the time synchronization error, especially for the channel with strong frequency selectivity.

**Figure 14:**
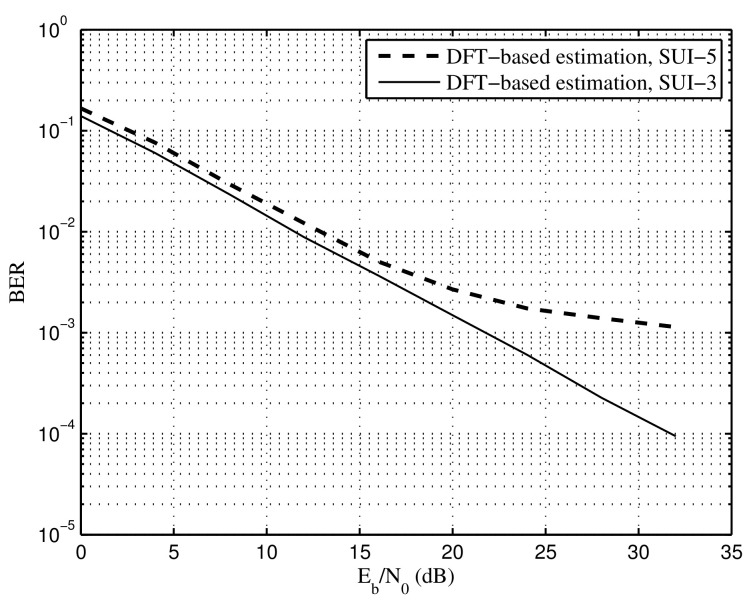
BER of the proposed DFT-based method, time synchronization error $L_{e}=10\;\mu$s.

Figure 14 shows BER of the proposed DFT-based method under SUI-3 and SUI-5, respectively, in which the time synchronization error is set to $L_{e}=10\;\mu$s. For the CP length, 0.25 T = 16.7 μs, 16.7 μs − 10 μs > 0.9 μs and 16.7 μs − 10 μs < 10 μs. Note that 0.9μs is the maximum channel delay of SUI-3 and 10μs is the maximum channel delay of SUI-5. According to the analysis in Section 3, the proposed SR-OFDM will suffer from the performance floor under the SUI-5 channel due to the interference among symbols, while there is no performance floor under the SUI-3 channel, which is verified in Figure 14.

As a remark, the CP overhead of the proposed SR-OFDM is 1/R of the classical OFDM. When *R* is set to 1, the proposed SR-OFDM is fully equivalent to OFDM. To highlight the contribution of this paper, we compare the proposed scheme with [12,13] as shown in Table 4. The proposed SR-OFDM does not have interference among symbols, and it is obvious that the SR-OFDM could also perform well at the region of high SNR compared to [12]. In addition, when *R* is small, the increase of complexity of the proposed SR-OFDM is acceptable in practice compared to [13].

## 6. Conclusions

In this paper, a novel waveform, SR-OFDM, was proposed based on the concept of symbol repetition. Compared with the classical OFDM, the CP overhead of the proposed SR-OFDM could be greatly reduced at the cost of an acceptable increased complexity. It was demonstrated that the multi-path channel can be efficiently overcome with a simple single-tap equalizer, even under a time synchronization error. The robustness to the time synchronization error would make the proposed SR-OFDM favorable in practice, since it could release the dependence on time synchronization accuracy. The performance of the proposed SR-OFDM has been verified by numerical simulations. As a remark, due to the difference to OFDM regarding the symbol demodulation, the combination of SR-OFDM and MIMO remains an important issue for one of our future works.

## Figures and Tables

**Table 1:** Some notations in this paper.

$j=\sqrt{-1}$	imaginary unit
*M*	subcarrier number
*R*	number of symbol repetition
$d_{m,r}$	transmitted symbol
r∈[0,R−1]	index of branch in SR-OFDM, not the index of repetition
m∈[0,M−1]	index of subcarrier
$L_{h}$	timing synchronization error
$y_{mR+r}$	the received signal after DFT, $y_{mR}\;↔\;d_{m,0}$, $y_{m_{0}R+r}\;↔\;d_{m,r},\;\;r\neq 0$

**Table 2:** Complexity comparison ofthe proposed SR-OFDM and the classical OFDM.

Multiplications	OFDM(*R* Symbols)	SR-OFDM
Transmitter	$RM\log_{2}\left(M\right)$	$RM\log_{2}\left(M\right)+R^{2}M$
Receiver	$RM\log_{2}\left(M\right)$	$\left(2R-1\right)RM\log_{2}\left(RM\right)+\left(R-1\right)RM$

**Table 3:** Profiles of SUI-3 and SUI-5 channel models.

Channel	SUI-3	SUI-5
Sampling Frequency (MHz)	30.72	30.72
Number of Paths	3	3
Power Profile (in dB)	0, −5.0, −10.0	0, −5.0, −10.0
Delay Profile (μs)	0, 0.4, 0.9	0, 4, 10
Frequency selectivity	low	high

**Table 4:** Comparison of different CP reduction schemes.

Element	Attribute	Characteristic
Ov-OFDM [12]	Propose a CP-free method for OFDM systems based on overlapping MMSE.	Remaining interference, applicable to low SNR.
Vector OFDM [13]	Propose a unit matrix for precoding multiple symbols so that multiple symbols only include one CP.	No inter-symbol interference, high complexity compared to other two methods.
SR-OFDM	Propose a novel waveform to make multiple symbols share one CP based on the concept of symbol repetition.	No inter-symbol interference, acceptable increased complexity.

## Data Availability

Not applicable.
